# Hijacked by the Feed: Social Media Neuroengineering-Induced Digital Anhedonia

**DOI:** 10.7759/cureus.83256

**Published:** 2025-04-30

**Authors:** Shaheen E Lakhan

**Affiliations:** 1 Bioscience, Global Neuroscience Initiative Foundation, Miami, USA; 2 Neurology, Andrew Taylor (AT) Still University School of Osteopathic Medicine in Arizona, Mesa, USA; 3 Neurology, Morehouse School of Medicine, Atlanta, USA; 4 Neurology, Western University of Health Sciences, Pomona, USA

**Keywords:** adolescent brain development, attention economy, digital anhedonia, digital overstimulation, emotional dysregulation, functional neuroimaging, neuroplasticity, prescription digital therapeutics, reward desensitization, social media addiction

## Abstract

Social media platforms have evolved from communication tools into hyperstimulating digital environments that directly engage reward and attention networks in the brain. Emerging neuroimaging studies reveal that heavy use, particularly among adolescents, is linked to functional and structural changes in regions governing emotional regulation, impulse control, and social cognition. These neural patterns resemble those seen in addiction, attention-deficit/hyperactivity disorder, and mood disorders. However, clinical medicine has been slow to respond. This editorial argues that we must reframe social media overuse as a neurologically mediated risk factor rather than just a behavioral concern. In addition to addiction-like engagement, a new affective pattern is emerging: “digital anhedonia,” the diminished ability to find pleasure in real-world experiences after prolonged digital saturation. As a neurologist and neuroscientist who develops smartphone-based applications for therapeutic benefit, I have seen both the healing and the harm these technologies can cause. It is time to recognize, monitor, and mitigate the neurobiological consequences of digital overstimulation and reward desensitization.

## Editorial

The phrase "social media addiction" is often dismissed as cultural hyperbole. However, the mounting neurobiological evidence suggests otherwise. Platforms like TikTok, Instagram, and YouTube are not merely affecting behavior; they are shaping brain development, especially in adolescents. Unlike previous generations, today’s youth are raised in an environment of continuous digital stimulation. Notifications, algorithmic content delivery, and dopamine-triggering feedback loops form the backbone of social media design. These elements are not passive; they are active agents of neural remodeling.

As physicians, we screen for lead exposure, sleep deprivation, and trauma. However, few of us ask about digital habits, despite their growing imprint on the brain. This editorial argues for a paradigm shift: social media overuse should be viewed not as a behavioral quirk but as a neurodevelopmental risk factor that requires clinical attention.

The brain on social media

Functional MRI studies show that heavy social media users exhibit increased activity in the ventral striatum and amygdala, regions associated with reward anticipation and emotional salience [1]. A 2023 longitudinal study found that habitual checking of social platforms over time predicted heightened neural responsiveness to social feedback cues [2]. These brain changes are not transient. Neuroplasticity during adolescence means that circuits reinforced through repetition, especially those related to rewards, become deeply ingrained. Repeated stimulation may condition the brain to favor digital experiences over real-world ones, leading to behavioral dependence. Meanwhile, underactivation in executive control areas such as the dorsolateral prefrontal cortex may impair impulse control and attention regulation.

These adaptations mirror findings in substance use disorders and gambling addiction, where natural rewards are devalued relative to synthetic, high-dopamine stimuli. The brain does not discriminate between biochemical and behavioral reinforcers. It simply reinforces what is immediate, novel, and rewarding. In this light, social media is not just habit-forming; it is neuroadaptive.

Digital anhedonia: a new affective landscape

Beyond compulsive use, clinicians are witnessing a subtler syndrome: individuals who are not clinically depressed but no longer derive pleasure from everyday activities. We term this phenomenon "digital anhedonia," a selective blunting of reward responses to analog stimuli following chronic digital overexposure. Unlike classic anhedonia in depression, these individuals may report stable energy, motivation, and sleep. They do not meet Diagnostic and Statistical Manual of Mental Disorders (DSM) criteria for a mood disorder but describe feeling "bored" by in-person events or hobbies and "flat" in the absence of screen-based stimulation.

Neuroimaging studies support this differentiation. Frequent social media users exhibit diminished activation in the nucleus accumbens and orbitofrontal cortex during non-digital reward tasks [3]. These regions are critical to encoding reward value and subjective pleasure. In effect, the brain becomes less sensitive to natural rewards, much like tolerance in addiction. Adolescents are particularly vulnerable due to the plasticity of their reward systems and their heightened sensitivity to social validation.

Behavioral and emotional fallout

Clinicians increasingly encounter teens and young adults reporting irritability, poor concentration, disrupted sleep, or social withdrawal [4]. While these may be interpreted as anxiety, attention-deficit/hyperactivity disorder, or subclinical depression, a unifying feature is often overlooked: digital overstimulation and its downstream neurocognitive impact. Affective flattening, decision fatigue, and avoidance of effortful tasks may result not from inherent pathology but from mismatched neural expectations set by the immediacy and variability of digital feedback loops.

Moreover, emotional dysregulation is amplified by algorithmic exposure to emotionally charged content, outrage, fear, and comparison. This contributes to stress reactivity and mood volatility. Research has shown that high social media exposure is correlated with increased amygdala reactivity and reduced prefrontal inhibition, mechanisms also found in emotional lability disorders.

Cognitive cost is another concern. Continuous partial attention, compulsive checking, and multitasking behaviors impair working memory and diminish attentional stability. These changes are reversible with behavioral interventions, but only if recognized.

Clinical blind spots and diagnostic oversight

Despite its widespread impact, digital anhedonia is not formally recognized in the DSM-5 or the International Classification of Diseases (ICD)-11 taxonomies. As a result, it is rarely screened for, often misdiagnosed, and nearly always undertreated. Digital behavior is generally excluded from psychiatric history unless associated with overt addiction or cyberbullying. This is a critical gap.

We advocate for including digital exposure patterns in psychiatric and neurological assessments, screening for quantity (e.g., screen time) and quality (e.g., type of content, context of use, emotional consequences). Clinicians should ask: Does the patient still find pleasure in offline activities? Is the capacity for reward linked to digital feedback? Are baseline reward thresholds shifting?

Toward intervention and recovery

Intervention strategies must address both behavioral habits and neuroplastic potential. "Digital hygiene" should become a standard counseling component, limiting non-essential screen use, promoting device-free environments, and encouraging analog socialization. For moderate cases, structured digital detoxes can help restore sensitivity in the reward system.

Behavioral activation strategies, long used in treating depression, can be adapted to favor offline, effortful activities with delayed gratification (e.g., taking nature walks, working on creative projects, engaging in service-based activities). Therapeutic journaling or cognitive-behavioral therapy can reframe distorted beliefs about pleasure, novelty, and validation.

Emerging digital therapeutics may also offer part of the solution. Paradoxically, apps grounded in evidence-based principles such as mindfulness, cognitive training, and guided behavior change can help users re-regulate their digital behaviors. The key distinction lies in whether the digital therapeutic is designed for pure engagement versus well-being and healthy outcomes. In other words, is the goal to capture attention or to restore it? Platforms that measure and adapt to emotional and cognitive states in real-time, rather than amplifying them, promise to recalibrate reward pathways and improve self-regulation. As with any clinical tool, intent and integrity of design determine whether the outcome is healing or harm.

Education systems must play a role. Schools should incorporate digital literacy curricula beyond online safety to teach about neural adaptation, dopamine dynamics, and emotional regulation. Empowering youth to understand how their brains respond to tech may be the most effective prevention.

Ethical and societal implications

We must confront a broader ethical question: Should industries be allowed to deploy neurostimulation tools without being held accountable for their long-term neural impact? If medications require safety profiles and controlled trials, why not interfaces designed to trigger dopamine surges?

Policy considerations include warning labels, recommended usage caps for minors, transparency in algorithmic intent, and integration of mental health impact assessments in app store approval processes. The responsibility should not fall solely on individuals; systems must evolve to protect developing brains.

Conclusions

Digital anhedonia may be the first affective disorder of the attention economy, born not of intrinsic psychopathology but of environmental neuroengineering. Its emergence demands that we evolve our diagnostic models, clinical training, and public health strategies.

As a neurologist and neuroscientist who develops smartphone-based therapeutics, I believe we must channel the same neuroplastic power that can harm into tools that heal. The human brain is adaptive, but it requires our stewardship. Our patients are not disengaged by choice; they are disengaged by design. The time to act is now.
